# *Toxoplasma gondii* infection disrupts secondary bile acid transformation in feline gut microbiota

**DOI:** 10.1128/aem.00674-26

**Published:** 2026-09-11

**Authors:** Lu-Yao Tang, He Zhang, Ji-Xin Zhao, Ying-Qian Gao, Xuancheng Zhang, Shi-Chen Xie, Zhi Zheng, Xing-Quan Zhu, Xiao-Xuan Zhang, Changyou Xia, Rui Liu, He Ma

**Affiliations:** 1 College of Veterinary Medicine, Qingdao Agricultural University 98431, Qingdao, Shandong, China; 2 State Key Laboratory of Animal Disease Control and Prevention, Harbin Veterinary Research Institute, Chinese Academy of Agricultural Sciences 111613, Harbin, China; 3 State Key Laboratory for Zoonotic Diseases, Key Laboratory for Zoonosis Research of the Ministry of Education, Institute of Zoonosis, and College of Veterinary Medicine, Jilin University 12510, Changchun, Jilin, China; 4 Laboratory of Parasitic Diseases, College of Veterinary Medicine, Shanxi Agricultural University 644568, Taigu, Shanxi, China; Centers for Disease Control and Prevention, Atlanta, Georgia, USA

**Keywords:** bile acid transformation, feline gut microbiota, metagenomic analysis, *Toxoplasma gondii*

## Abstract

Bile acid (BA) transformation relies on gut microbiota and is vulnerable to *Toxoplasma gondii* infection, yet feline microbial BA-transforming capacity upon toxoplasmosis remains unclear. Here, we constructed a catalog of 2,474 nonredundant feline gut microbial genomes and integrated serum metabolomic data to verify BA transformation alterations. The results revealed that the feline gut microbiome harbored widespread genetic potential for BA transformation but lacked a complete 7α-dehydroxylation pathway due to the absence of the key gene *baiE*. The BA transformation-related genomes (2,045 in total) were predominantly from the phyla Bacillota_A and Actinomycetota, among which only 37 encoded *baiB*, all belonging to Bacillota_A. The distribution of BA transformation-related genes varied across intestinal regions: genes encoding 7α-HSDH were primarily enriched in the small intestine, whereas genes encoding 3α-HSDH, *baiCD*, and *baiH* were more abundant in the large intestine. Additionally, the abundance of genes encoding BSH and 3α-HSDH increased significantly in the small intestine on day 3 post-infection, accompanied by increases in the phylum Bacillota_C and genera such as *Blautia_A*, *Enterococcus_E*, and *Ligilactobacillus*. Serum metabolomics revealed a significant increase in cholesterol levels post-infection, supporting the impact of *T. gondii* infection on intestinal BA transformation. These findings illustrated that the feline gut microbiota played an important role in BA transformation and that *T. gondii* infection disrupted the microbial potential for secondary BA transformation. This study provided new insights into gut microbiota-associated metabolic perturbations during feline toxoplasmosis.

**IMPORTANCE:**

Bile acid (BA) transformation plays a critical role in host metabolism and immune regulation. Although studies on BA transformation are increasing, the capacity for BA transformation within the feline gut microbiota and the impact of *Toxoplasma gondii* infection on this capacity remain unclear. To bridge this gap, we constructed a catalog of 2,474 nonredundant feline gut microbial genomes and integrated serum metabolomic data to verify BA transformation alterations. Our findings revealed that the feline gut microbiome lacked a complete 7α-dehydroxylation pathway, and the specific functions involved in BA transformation may differ between the small and large intestines. Furthermore, integrated metagenomic and serum metabolomic analyses suggested that *T. gondii* infection disrupted BA transformation capacity in the small intestine. This study provided new insights into gut microbiota-associated metabolic perturbations during feline toxoplasmosis.

## INTRODUCTION

Bile acids (BAs) are cholesterol derivatives synthesized by the liver. They serve as potent metabolic and immune signaling molecules and are involved in lipid absorption, cholesterol homeostasis, and enterohepatic axis signaling regulation (1, 2). Host hepatocytes utilize cholesterol to synthesize primary BAs, which are subsequently chemically modified by the intestinal microbiota into secondary BAs (3). This chemical modification occurs through four main transformation pathways: deconjugation, dehydroxylation, oxidation, and epimerization (4). The key enzymes and corresponding genes involved in these pathways have been well characterized: deconjugation is mediated by bile salt hydrolase (BSH); 7α-dehydroxylation is mediated by the *bai* operon; and oxidation and epimerization are mediated by hydroxysteroid dehydrogenases (HSDHs) (5). The composition and function of the BA pool are influenced by multiple factors, including host genetics, diet, and the gut microbiota, with the gut microbiota playing a pivotal role (6–8). The enzymatic activities of different microbial communities determine the unique composition and function of the BA pool (9, 10), and the physicochemical environment of different intestinal regions further regulates BA transformation by selectively shaping the composition of the microbial community (11). Therefore, there is a close interaction between the gut microbiota and BAs (12). Although there have been increasing studies on BAs, research on the role of the gut microbiota in BA transformation in felines remains limited.

Notably, numerous studies have demonstrated that parasite infection profoundly impacts host immunity and metabolic homeostasis and significantly alters the composition and function of the gut microbiota (13–16). During *Toxoplasma gondii* infection, changes in gut microbial diversity and the abundance of specific functional microbial groups may indirectly affect the gut microbiota’s capacity for secondary BA transformation (16). In response to *T. gondii* infection, the host undergoes metabolic reprogramming, in which cholesterol metabolism and BA signaling pathways play key roles in immune modulation and cellular defense (17–19). Therefore, the alterations in host cholesterol metabolism and the structural and functional changes in the gut microbiota induced by *T. gondii* infection may influence BA transformation, thereby affecting the host’s immunometabolic homeostasis. However, it remains unclear how the host regulates cholesterol levels and how the BA transformation capacity of the gut microbiota changes during *T. gondii* infection.

To address the above questions, we analyzed available metagenomic data sets and integrated previously published serum metabolomic data to evaluate the impact of *T. gondii* infection on the feline gut microbiota and secondary BA transformation. We characterized the feline gut microbiome, focusing on key genes involved in BA transformation, and analyzed serum cholesterol levels. This study elucidated the BA transformation capacity of feline gut microbiota and the impact of *T. gondii* infection on this process, providing new insights into gut microbiota-related metabolic dysregulation during toxoplasmosis and laying a theoretical foundation for future research on host-parasite-gut microbiota interactions.

## RESULTS

### Profiling of feline gut microbiome

**Fig 1:**
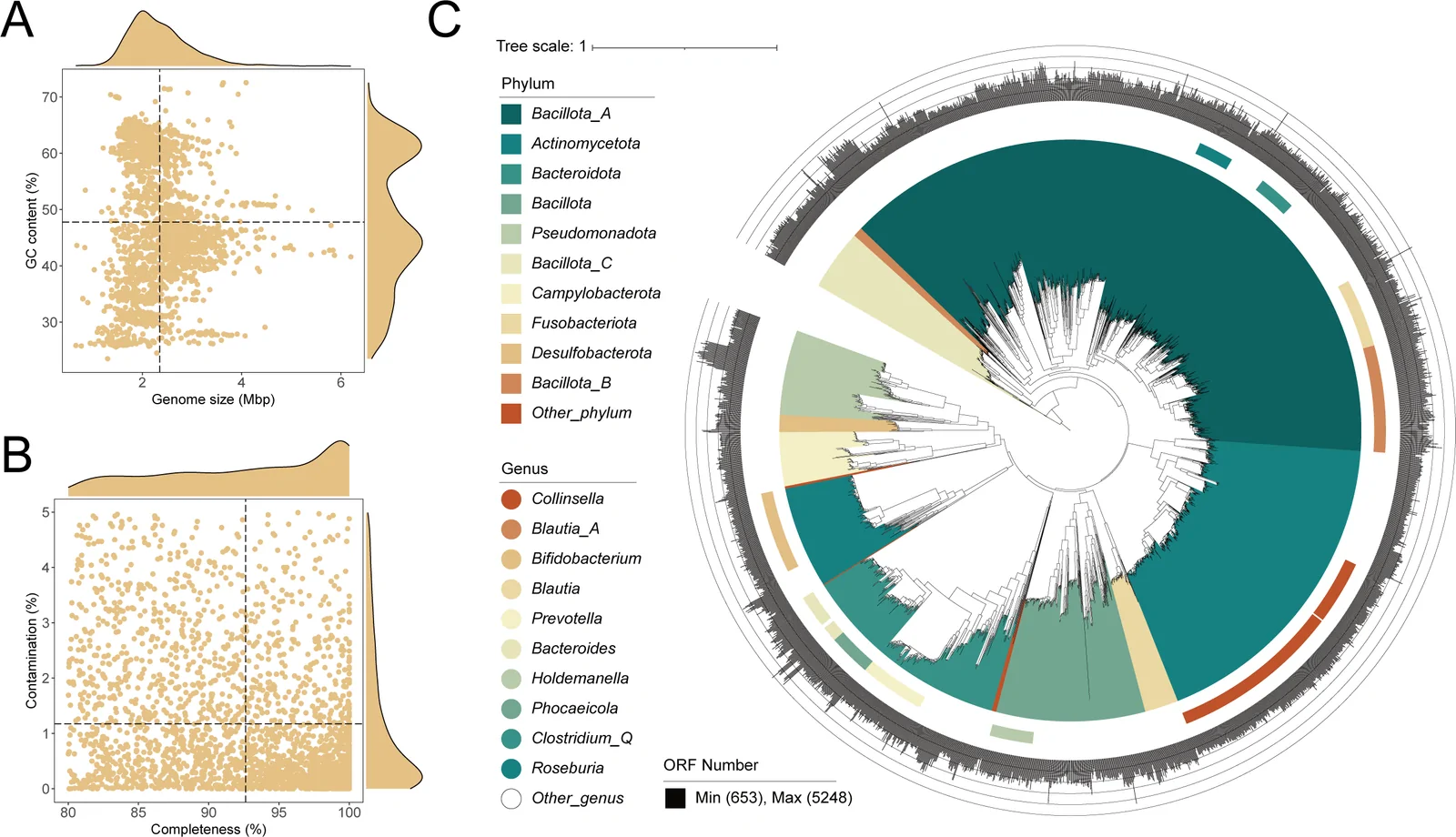
Summary of collected feline intestinal genomes. (**A**) The genome size (in megabase pairs, Mbp) and GC content of genomes. (**B**) The completeness and contamination rate of genomes. (**C**) A phylogenetic tree of 2,474 genomes. The clades are colored according to the phylum. The strip chart indicates the genus-level affiliations of genomes. The bar chart shows the number of open reading frames (ORFs) in each genome.

A total of 2,474 nonredundant representative genomes were analyzed in this study. These genome sizes ranged from 0.66 to 6.20 Mbp (average 2.35 Mbp), with the GC content spanning 23.49% to 72.53% (average 47.76%) (Fig. 1A). The genomes exhibited completeness ranging from 80.01% to 100% (average 92.63%) and contamination ranging from 0% to 4.99% (average 1.17%) (Fig. 1B). Among them, 1,613 genomes (65.20%) met high-quality standards (completeness ≥90% and contamination <5%). The number of open reading frames (ORFs) per genome varied from 653 to 5,248 (average 2,203). For taxonomic annotation analysis, 2,474 genomes were classified within bacterial lineages, covering 17 phyla, 21 classes, 48 orders, 90 families, and 279 genera. The vast majority of genomes were assigned to Bacillota_A (*n* = 988, 39.94%), followed by Actinomycetota (*n* = 598, 24.17%) and Bacteroidota (*n* = 298, 12.05%). At the class level, the most abundant was Clostridia (*n* = 988, 39.94%), followed by Coriobacteriia (*n* = 454, 18.35%) and Bacteroidia (*n* = 298, 12.05%). At the genus level, the genomes spanned a wide taxonomic diversity; the most abundant genera were *Collinsella* (*n* = 303, 12.25%), *Blautia_A* (*n* = 139, 5.62%), and *Bifidobacterium* (*n* = 106, 4.28%) (Fig. 1C and Table S1). Additionally, a microbial gene catalog comprising 5,449,269 genes was constructed by gene prediction. These genome and gene catalogs served as important resources for studying the classification and function of the feline gut microbiome.

### Catalog of genomes related to BA transformation

**Fig 2:**
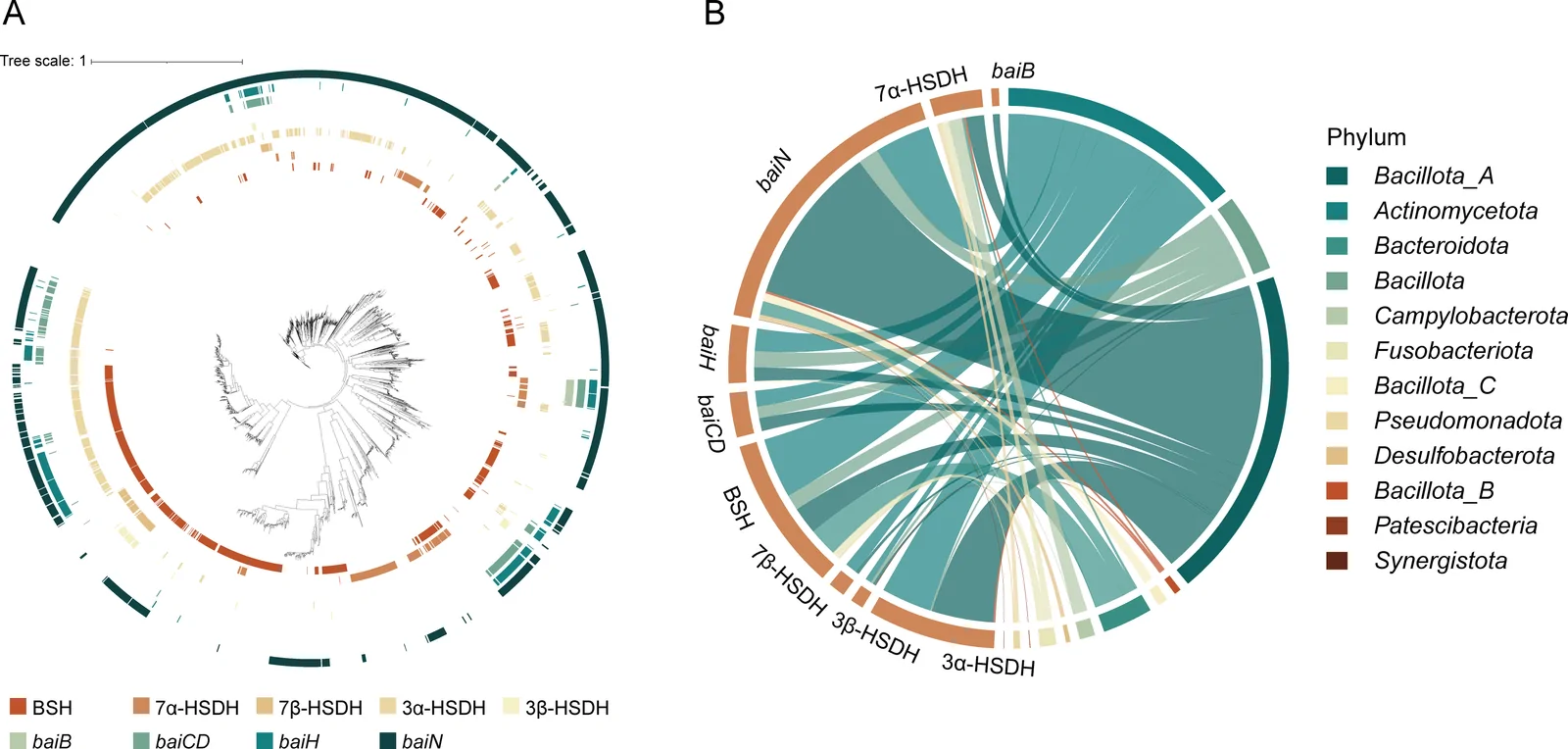
Summary of genomes related to bile acid (BA) transformation. (**A**) The presence (colored) or absence (uncolored) of the encoding capacity of enzymes related to BA transformation in the genome, with each color representing a specific enzyme. (**B**) Chord diagram showing the taxonomic (phylum-level) distribution of genomes encoding each enzyme.

Based on KEGG functional annotation, 2,045 out of 2,474 genomes were predicted to contain at least one KO associated with secondary BA transformation. Analysis of these 2,045 genomes revealed that 950 genomes carried one KO, 636 genomes carried two, 455 genomes carried three to five, and four genomes carried six (Table S2). The number of genomes related to each enzyme or *bai* gene was as follows: *baiN* (1,446 genomes), BSH (766 genomes), 3α-HSDH (631 genomes), *baiH* (288 genomes), 7α-HSDH (258 genomes), *baiCD* (220 genomes), 7β-HSDH (83 genomes), 3β-HSDH (64 genomes), and *baiB* (37 genomes) (Fig. 2A). Notably, none of the genomes were related to the key enzyme *baiE*, indicating that a complete 7α-dehydroxylation pathway required for secondary BA transformation may not be present in the feline gut microbiome.

Taxonomic annotation revealed that these genomes encompassed 12 phyla, 13 classes, 34 orders, 64 families, and 226 genera (Table S2) The presence of BA-related KOs in the feline gut was mainly attributed to Bacillota_A and Actinomycetota. Specifically, 3α-HSDH, 7β-HSDH, *baiN*, and *baiCD* were predominantly associated with genomes from these two phyla: Bacillota_A contributed 53.09%, 18.07%, 60.03%, and 31.36%, respectively, while Actinomycetota contributed 43.42%, 81.93%, 20.95%, and 39.09%, respectively. *baiB* was exclusively found in genomes from Bacillota_A (100%). 3β-HSDH and *baiH* were mainly present in genomes from Actinomycetota and Bacillota, with Actinomycetota contributing 50.00% and 42.01% and Bacillota contributing 25.00% and 31.25%, respectively. 7α-HSDH was primarily associated with genomes from Bacillota_A (37.21%) and Campylobacterota (29.07%), while BSH was mainly related to genomes from Actinomycetota (42.82%) and Bacteroidota (20.76%) (Fig. 2B). These results indicated that secondary BA transformation was accomplished through the collective collaboration of the gut microbial community.

### Comparison of gut microbial genomes involved in BA transformation between felines and other host species

**Fig 3:**
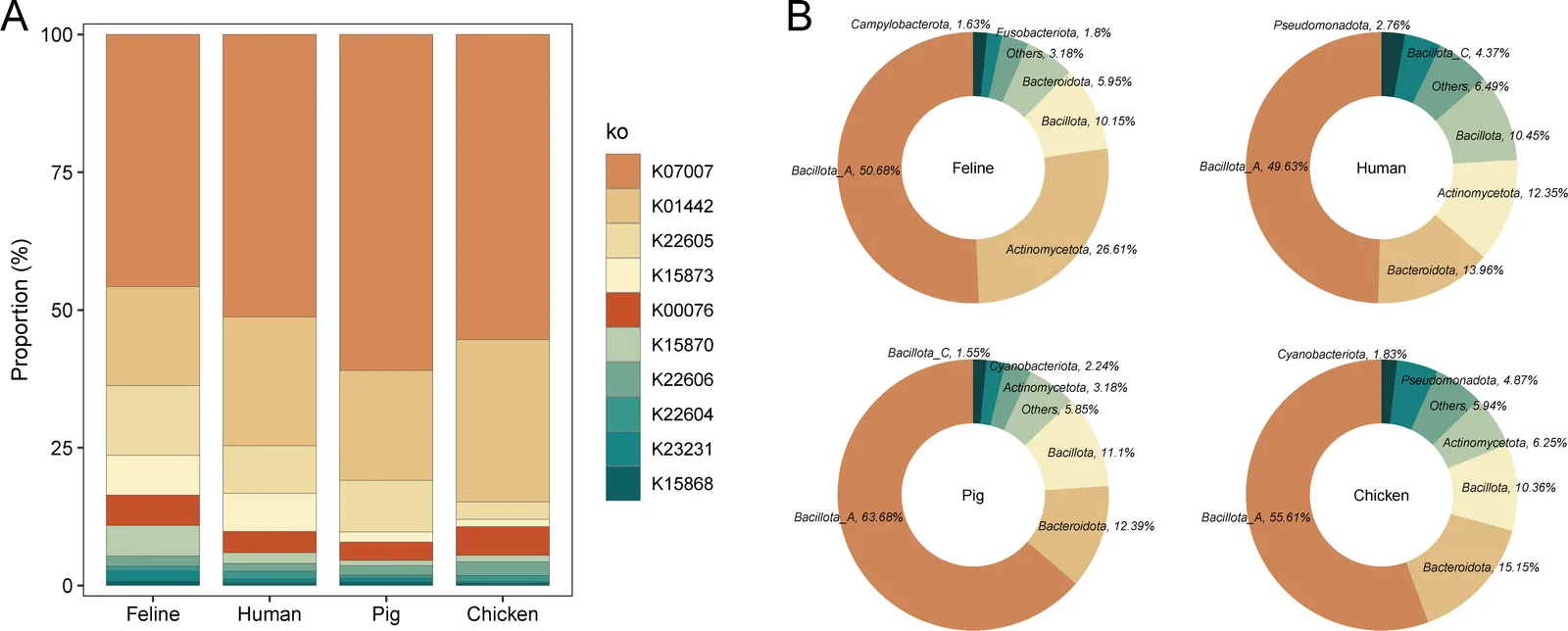
Summary of the intestinal genomes related to bile acid (BA) transformation in different host species. (**A**) Proportion of BA transformation-related KEGG orthologs across four host species. (**B**) Phylum-level composition of BA-related genomes across four host species.

To elucidate the specificity of the feline gut microbial genomes involved in BA transformation, we compared the 2,045 BA-related genomes from cats with those from other host species: humans (2,294 metagenome-assembled genomes [MAGs]), pigs (1,411 MAGs), and chickens (2,113 MAGs). Annotation analysis showed that 1,741 human MAGs, 1,162 pig MAGs, and 1,621 chicken MAGs were also involved in BA transformation. Among the four host species, the proportions of *baiN* (K07007) and BSH (K01442) were the highest (Fig. 3A). At the phylum level, Bacillota_A was the primary contributor to BA-related MAGs across all four host species. Actinomycetota served as a secondary contributor in the feline gut microbiota, accounting for 26.61%, whereas Bacteroidota was the secondary contributor in the other three host species, accounting for 13.96%, 12.39%, and 15.15% in the human, pig, and chicken gut microbiota, respectively (Fig. 3B and Table S3) These findings revealed host species-specific differences in the primary genomic contributors to BA transformation.

### Distribution and regional differences of BA-related genes

**Fig 4:**
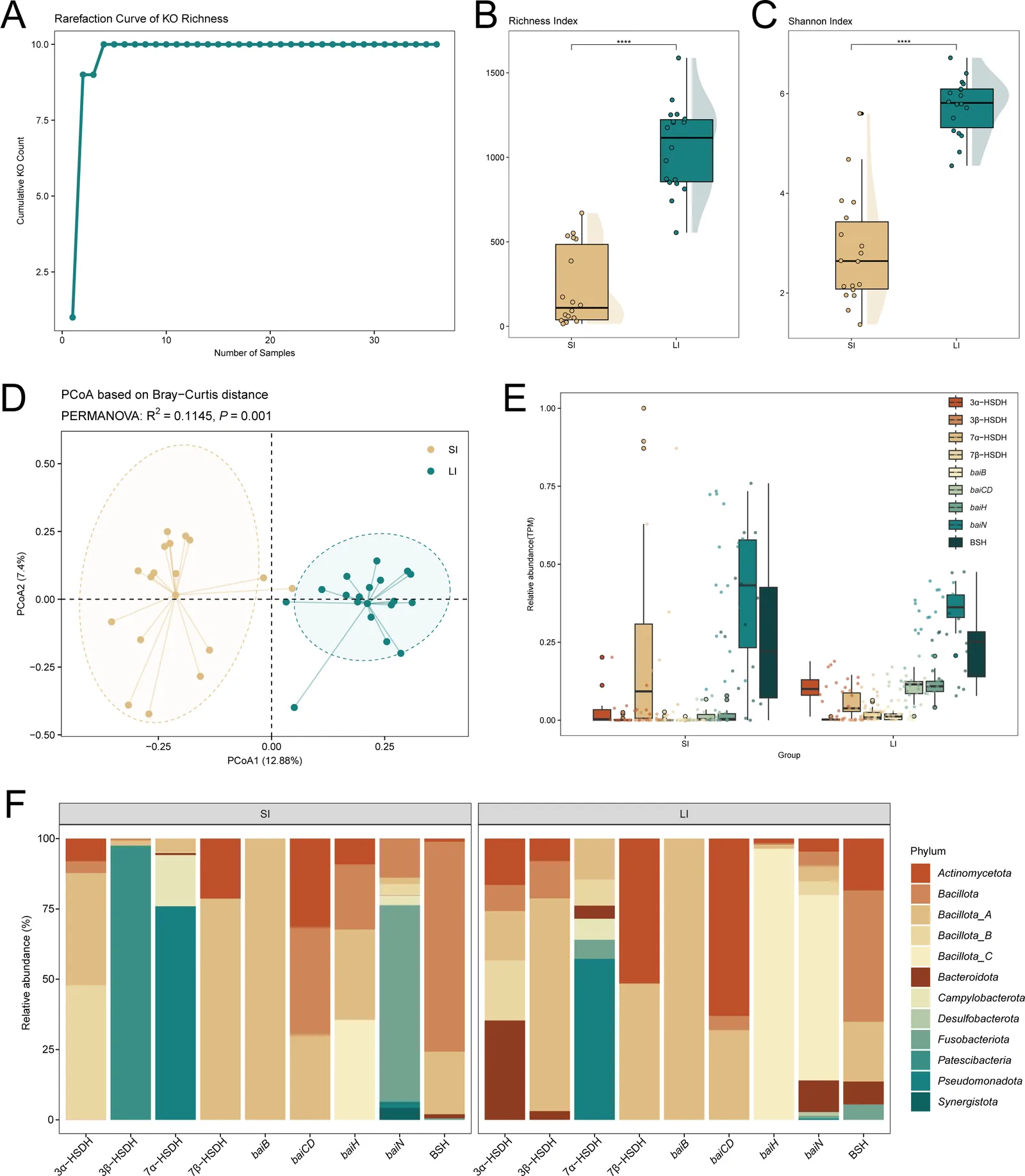
Microbial gene abundance, diversity, and community structure related to bile acid (BA) transformation in different intestinal regions. (**A**) Rarefaction curve analysis illustrating the relationship between gene abundance and sample size. (**B and C**) Boxplots displaying the Richness and Shannon indices in the small intestine (SI) and large intestine (LI). (**D**) Principal coordinates analysis (PCoA) scatter plot illustrating the compositional differences in the intestinal microbiota among different intestinal regions. (**E**) Relative abundance of microbial genes associated with BA transformation in the small intestine (SI) and large intestine (LI). (**F**) Bar graphs showing the phylum-level distribution of gene sets related to BA transformation.

We analyzed the distribution of BA-related genes in the gut microbiota of felines across different intestinal regions and among different taxa. As the number of samples increased, the rarefaction curve for KO richness approached saturation, indicating that our analysis fully covered the KO genes related to secondary BA transformation in the feline gut microbiome (Fig. 4A). The alpha and beta diversity indices were used to evaluate the distribution differences of these functional genes among different intestinal regions. The alpha diversity measured by Richness and Shannon indices indicated that the diversity and richness of BA-related genes were significantly higher in the large intestine (LI) than in the small intestine (SI) (*P* < 0.05) (Fig. 4B through C). Principal coordinates analysis (PCoA) based on Bray-Curtis distance showed a significant difference in the composition of BA-related genes between the large and small intestines (*R*² = 0.1145, *P* < 0.05) (Fig. 4D).

Analysis of the relative abundance of genes associated with BA transformation showed that genes related to *baiN* and BSH were abundant in both the small and large intestines, whereas genes associated with 3β-HSDH, 7β-HSDH, and *baiB* were present at low abundance. Meanwhile, the relative abundance of genes related to 7α-HSDH was higher in the small intestine than in the large intestine, whereas the relative abundances of genes associated with 3α-HSDH, *baiCD*, and *baiH* were lower in the small intestine than in the large intestine (Fig. 4E). We also performed taxonomic analysis of BA-related genes across different intestinal regions. At the phylum level, the distribution patterns differed between the small and large intestines. For instance, Fusobacteriota was the most abundant contributor to genes encoding *baiN* in the small intestine, whereas Bacillota_C dominated in the large intestine. For genes encoding BSH, the most abundant phylum in both the small and large intestines was Bacillota, followed by Bacillota_A (Fig. 4F).

### *T. gondii* infection disrupts BA transformation

**Fig 5:**
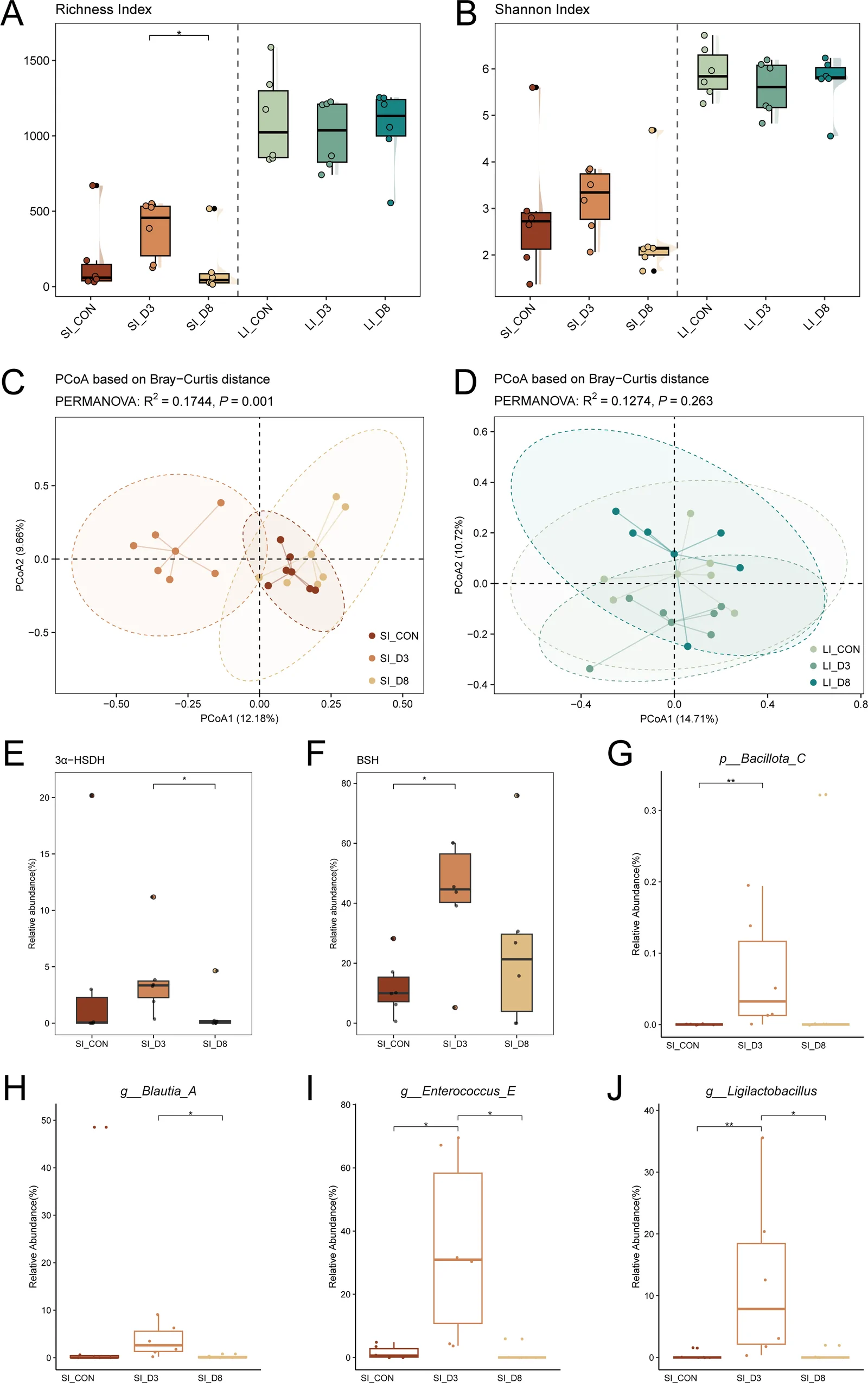
Analysis of bile acid (BA) transformation-related genes across intestinal regions and infection stages. (**A and B**) Richness and Shannon diversity indices of BA transformation-related genes in the small intestine (SI) and large intestine (LI) across different infection stages. (**C and D**) Principal coordinates analysis (PCoA) scatter plot illustrating the microbial community structure at different infection stages in different intestinal regions. (**E and F**) Boxplots showing the relative abundance of genes encoding 3α-HSDH and BSH in the small intestine before and after *T. gondii* infection. (**G**) Boxplot showing the relative abundance of the small intestinal microbiota at the phylum level before and after *T. gondii* infection. (**H–J**) Boxplots showing the relative abundance of the small intestinal microbiota at the genus level before and after *T. gondii* infection. Statistical significance was determined using the Wilcoxon rank-sum test (* *P* < 0.05; **<*I>P* < 0.01; ***<*I>P* < 0.001). *Abbreviations:* SI, small intestine; LI, large intestine; CON, control group (pre-infection); D3, day 3 post-infection; D8, day 8 post-infection.

To investigate the impact of *T. gondii* infection on BA transformation in the feline gut microbiota, we reanalyzed metagenomic data collected before and after infection. Alpha diversity analysis revealed a significant increase in the richness of BA-related genes only in the small intestine on day 3 post-infection, with no significant changes observed elsewhere (Fig. 5A through B). PCoA based on Bray-Curtis distance further indicated that the composition of BA-related genes was primarily altered in the small intestine following infection (Fig. 5C through D). These findings suggested that the small intestine responded more sensitively to infection-induced changes in BA transformation, whereas the large intestine remained relatively stable.

We also analyzed changes in the relative abundance of genes related to various enzymes or *bai* genes in both intestinal regions. Results showed that only the relative abundance of genes encoding 3α-HSDH and BSH increased significantly in the small intestine on day 3 post-infection, while no significant changes were observed in other BA-related enzymes or *bai* genes (Fig. 5E through F). Notably, following *T. gondii* infection, the relative abundance of genes encoding 3α-HSDH and BSH exhibited a trend of initially increasing and then decreasing, suggesting that the impact of *T. gondii* infection on the BA transformation potential of the small intestinal microbiota may be time-dependent. To identify the microbial species responsible for these alterations, taxonomic analysis was performed on BA-related genes. At the phylum level, Bacillota_C showed a significant increase in the small intestine on day 3 post-infection (Fig. 5G). Among the top 10 genera with the highest relative abundance, the following exhibited significant changes: *Blautia_A*, *Enterococcus_E*, and *Ligilactobacillus* significantly increased in the small intestine on day 3 post-infection (Fig. 5H through J). These results indicated that specific microbial groups may be involved in the alteration in BA transformation potential during the early stages of *T. gondii* infection.

To further explore the effects of *T. gondii* infection on BA synthesis and transformation, we integrated the findings from serum metabolomic analysis of the same cats. The results revealed that cholesterol levels significantly increased after infection compared to pre-infection levels (*P* < 0.05, Table 1), indicating that *T. gondii* infection may affect host cholesterol metabolism and be associated with alterations in gut microbiota-mediated BA transformation.

**TABLE 1:** Analysis of differential serum metabolites after *T. gondii* infection^*a*^

Comparison	Metabolite	VIP	FC	log2FC	*P*-value	Type
CON vs D3	Cholesterol	1.61523	92.707	6.53461	0.01759	Up
CON vs D8	Cholesterol	1.9843	67.6656	6.08035	0.00087	Up

^*a*^ VIP, variable importance in projection; FC, fold change; log2FC, log2-transformed fold change; CON, control group (pre-infection); D3, the D3 group (day 3 post-infection); D8, the D8 group (day 8 post-infection); up, significantly increased relative to CON.

## DISCUSSION

To investigate the functional role of the feline gut microbiota in secondary BA transformation, we constructed a catalog comprising 2,474 non-redundant representative genomes to illustrate its key functional characteristics. Taxonomic annotation revealed that the genomes encompassed broad taxonomic diversity, reflecting the diversity and complexity of the feline gut microbial community. The dominant classes within the feline gut microbiota in this study were Clostridia, Coriobacteriia, and Bacteroidia, which aligned with findings from Ricardo David Avellán-Llaguno et al. (20), who reported high relative abundances of Bacteroidia and Clostridia in the feline gut microbiota. Functional annotation identified 4,787 genes involved in BA transformation across 2,045 genomes, indicating that the feline gut microbiome harbors a widespread genetic potential for BA transformation. Notably, the number of genomes associated with different BA-related enzymes or *bai* gene clusters varied significantly. In this study, *baiN* (1,446 genomes) and BSH (766 genomes) were the two most prevalent. BSH, which catalyzes the deconjugation of primary BAs, is widely distributed across major bacterial and archaeal phyla, indicating that deconjugation was a common function in the feline gut microbiota (21). In contrast, *baiN*, a member of the *bai* gene cluster, was detected in a large number of genomes; however, other key *bai* genes were much less prevalent. For example, only 37 genomes were associated with *baiB*, which encodes BA-CoA ligase involved in substrate activation during the 7α-dehydroxylation process (22). Importantly, the key enzyme *baiE*, which catalyzes the essential dehydration step in the 7α-dehydroxylation pathway, was not detected in any genomes (23). These suggested that, although certain components of the *bai* gene cluster were present, a complete 7α-dehydroxylation pathway is likely absent in the feline gut microbiome. These findings indicated that BA transformation in cats was primarily mediated by widely distributed functions such as deconjugation and hydroxysteroid dehydrogenase-mediated modifications, whereas the capacity for complete secondary BA formation via 7α-dehydroxylation may be limited to a very small fraction of the microbiota or absent.

Comprehensive analysis revealed that Bacillota_A and Actinomycetota were the primary phyla associated with most key enzymes and *bai* gene clusters, suggesting that these two major phyla may play a dominant role in secondary BA transformation in the feline gut microbiota. Bacillota_A is a branch of the Bacillota phylum; previous studies have indicated that Bacillota (particularly Clostridia taxa) are the primary hosts for the 7α-dehydroxylation pathway and *bai* gene clusters (22, 24, 25), which is largely consistent with the findings of this study. Of particular note, genes encoding 7α-HSDH and BSH were derived from various microbial taxa, whereas 7β-HSDH was predominantly encoded by Actinomycetota, and *baiB* was exclusively derived from Bacillota_A. This distribution pattern indicated that BA transformation within the feline gut microbiota relied on an interactive network composed of diverse bacteria, which is significant for maintaining the metabolic homeostasis of the gut microecology (18, 26). It also suggested functional differentiation within the feline gut microbiota regarding secondary BA transformation, with the vast majority of microbiota participating only in preliminary transformation steps.

Comparative analysis of different host species showed that the proportions of BA-related MAGs in the gut microbial genomes of each host species were highly similar, indicating that BA transformation is a fundamental and essential function in the gut microbiota of different host species (27, 28). BSH was predominant in the gut microbiota of all four host species examined, further confirming its central role in microbial BA transformation. Taxonomic annotation results showed that the phylum Bacteroidota was a minor yet stable contributor in humans, pigs, and chickens, but its contribution was significantly lower in felines. This difference may be related to host genotype, diet, and intestinal environment (29–31).

By analyzing the microbiota from different intestinal regions of felines, we revealed significant differences in the composition and diversity of functional genes related to BA transformation between the small and large intestines. This region-specific distribution pattern of functional genes provided an important background for understanding the functional differences in BA-related enzymes across different intestinal regions. Further analysis of the abundance of BA-related genes uncovered distinct functional characteristics in different intestinal regions. Genes encoding BSH were highly abundant in both the small and large intestines, demonstrating its core role in BA transformation. Notably, genes encoding 7α-HSDH were enriched in the small intestine, suggesting that small intestinal microbiota may play a key role in the oxidation-reduction reactions of the 7α-hydroxy group of BAs, generating critical intermediate metabolites involved in subsequent secondary BA transformation (32). Conversely, genes encoding 3α-HSDH, *baiCD*, and *baiH* were primarily enriched in the large intestine, indicating that the large intestinal microbiota may be more inclined to undertake the intermediate steps of BA transformation, including partial 7α-dehydroxylation reaction and further structural modification of bile acid intermediates (33, 34). This functional distribution may have been formed by the selective shaping of the microbiota by the physical and chemical environments of different intestinal regions (e.g., pH, oxygen content, and BA concentration) (35).

In this study, alpha diversity and PCoA analyses collectively indicated that changes in BA-related genes induced by *T. gondii* infection were primarily concentrated in the small intestine. Significant changes in the diversity of genes and community structure in the small intestine on the third day post-infection occurred, while the large intestine remained relatively stable. This finding aligned well with the physiological characteristics and immune response patterns of different gut regions. The small intestine is the primary site for *T. gondii* invasion and replication, experiencing drastic local changes such as inflammation and mucosal damage. This disruption may directly and rapidly impact the resident microbiota, thereby driving functional shifts (36, 37). In contrast, the microbiota of the large intestine may exhibit stronger resistance to acute infections due to its higher complexity and stability (38).

The abundance of genes related to various enzymes or *bai* genes in different intestinal regions was analyzed, and it was found that the relative abundance of genes encoding 3α-HSDH and BSH in the small intestinal microbiota significantly increased after infection. This suggested an increase in the abundance of microbiota encoding these enzymes, which may enhance the potential for secondary BA transformation mediated by the small intestinal microbiota, thereby affecting the transformation process from primary BAs to secondary BAs. Although secondary BAs can inhibit BA synthesis via the farnesoid X receptor signaling pathway under homeostatic conditions, in the context of infection, this negative feedback may be modulated by inflammatory responses and metabolic reprogramming, thereby sustaining active hepatic BA synthesis. As cholesterol is the sole precursor for BA biosynthesis, the host may compensatorily upregulate cholesterol synthesis to meet the increased demand, resulting in elevated serum cholesterol levels (8, 17, 39, 40). These changes indicated that *T. gondii* infection may induce a coordinated response involving host metabolic regulation and functional remodeling of the gut microbiota (41). Further taxonomic analysis linked these changes to specific microbial taxa. The significant increase in the abundance of the phylum Bacillota_C and the genera *Blautia_A*, *Enterococcus_E*, and *Ligilactobacillus* indicated that these microbial taxa may be key participants involved in or influencing BA transformation during infection. Relevant studies have reported that *Ligilactobacillus* can encode BSH enzymes and participate in secondary BA transformation (42), supporting its potential functional importance. The results of this study demonstrated that *T. gondii* infection preferentially disrupted the BA transformation capacity of the small intestinal microbiota, accompanied by dynamic changes in key functional genes and specific microbial taxa.

## MATERIALS AND METHODS

### Experimental design

All the experimental data were derived from the study we published previously (43). The original experimental design and sample information are described below. Briefly, 18 4-month- to 5-month-old specific-pathogen-free cats (*Felis catus*) were randomly divided into three groups (*n* = 6 per group) based on the different *Toxoplasma gondii* stages they were infected with. Group CON served as the control group (pre-infection). Group D3 was infected with the asexual reproduction stage (day 3 post-infection), and group D8 was infected with the sexual reproduction stage (day 8 post-infection). Cats in the infected groups were orally inoculated with 800 cysts of the *Toxoplasma gondii* PRU strain (Genotype II), while cats in the control group received an equivalent volume of phosphate-buffered saline (PBS). Intestinal content samples from the small and large intestines were collected for metagenomic sequencing, and serum samples from the same animals were subjected to untargeted metabolomics analysis in the previous study.

### Public data collection

We retrieved six public metagenomic projects from Recherche Data Gouv (the French national research data repository): PRJNA758898, PRJEB9357, PRJEB4391, PRJNA944553, PRJNA908260, and PRJNA923753. Additionally, two projects (PRJNA961122 and PRJNA1237286) were obtained from the NCBI database. Since project PRJNA1237286 overlapped with the five public projects from the first database, we performed integration and deduplication to ensure the uniqueness of each sample. Ultimately, we retained intestinal genomes from three independent projects (PRJNA961122, PRJNA1237286, and PRJNA923753) and 444 metagenomic samples.

### Preprocessing and bioinformatic analysis

Raw sequencing data were subjected to quality control and filtering using FASTP (v0.23.0) (44) with the parameters “-q 20 -u 30 -n 5 -y -y 30 -l 90 -trim_poly_g.” High-quality reads were aligned to the host reference genome using Bowtie2 (v2.5.0) (45) to remove host-derived sequences. Metagenomic assembly of each sample was performed with MEGAHIT (v1.2.9) (46) to generate contigs, using the parameter “-k-list 21,41,61,81,101,121,141.” Reads were mapped to the assembled contigs using BWA-MEM (v0.7.17) (47) and converted to BAM format with SAMtools (v0.1.19) (48). Sequencing depth for contigs was calculated using the jgi_summarize_BAM_contig_depth script from MetaBAT2 (v2.12.1) (49). Binning of contigs was conducted with MetaBAT2 (v2.12.1) (49) using the parameters “-M 2000 -s 200,000 -saveCls -unbinned.” According to the above steps, a total of 22,197 genomes were retrieved in this study.

CheckM2 (v1.0.1) (50) was used to estimate the completeness and contamination of the genomes, and the following selection criteria were applied to eliminate bias: completeness ≥80%, contamination <5%, and a quality score (completeness – 5 × contamination) ≥50%, resulting in the retention of 10,684 genomes. The retained genomes were clustered and dereplicated using dRep (v3.4.5) (51) with the following parameters: -pa 0.90 -sa 0.99 -nc 0.30. Genomes >  99% average nucleotide identity (ANI) were grouped together, and the genome with the highest quality score in each group was considered as the representative genome. In total, 2,474 nonredundant representative genomes were obtained for use in this study. Additionally, open reading frames (ORFs) in these representative genomes were predicted using Prodigal (v2.6.3) (52). Taxonomic annotation was assigned with GTDB-tk (v2.3.2) (53), and a phylogenetic tree was constructed using PhyloPhlAn (v3.0.51) (54) and visualized with iTOL.

### Functional analysis of BA-related microbial genes

Functional annotations for protein-coding genes were obtained by aligning their sequences against the Kyoto Encyclopedia of Genes and Genomes (KEGG) database (55) using DIAMOND (v2.1.8.162) (56) and BLASTP (57). Based on previous studies (4, 58–60), 15 KEGG Orthology (KO) identifiers (e.g., K00076 and K01442) that correspond to the currently annotated genes encoding key enzymes involved in secondary BA transformation were selected for gene screening. These KOs correspond to key enzymes involved in the four main BA transformation pathways outlined in the introduction: deconjugation (BSH), oxidation/epimerization (7α-, 7β-, 3α-, and 3β-HSDHs), and 7α-dehydroxylation (members of the *bai* gene cluster, including *baiA, baiB, baiCD, baiE, baiF, baiH, baiI,* and *baiN*). For inter-sample comparison, Bowtie2 (v2.5.0) (45) was used to align high-quality sequencing reads from the 36 samples to the selected genes. The mapped read count of the gene was normalized to transcripts per million (TPM).

To enable functional comparison across host species, we included metagenome-assembled genomes (MAGs) from humans (61), pigs (62), and chickens (63). These data sets were collected and compiled by our research team from mainstream and publicly available sources and are all representative. All MAGs were processed using the same analytical pipeline as the feline gut microbial genomes described above. Genes predicted from these MAGs were subjected to the same KEGG annotation pipeline as the feline gut microbial genes to ensure comparability across host species.

### Serum metabolomics analysis

The serum metabolomic raw data adopted herein were generated in our previously published study (41). The experimental procedures for sample pretreatment and instrumental detection are briefly summarized below. Serum samples were subjected to protein precipitation using methanol/acetonitrile (1:1, vol/vol). After vortexing, cryo-sonication, and centrifugation, the supernatant was collected and evaporated in a vacuum concentrator. The dried extract was re-dissolved in 100 μL of 50% methanol supplemented with 5 ppm 2-chlorophenylalanine and centrifuged again. The clarified supernatant was subsequently used for LC–MS/MS analysis. An equal volume from each sample was pooled to prepare quality control (QC) samples for monitoring system stability. Metabolite detection was performed on a Thermo Vanquish UHPLC–Orbitrap Exploris 120 platform, with data-dependent acquisition (DDA) conducted in both positive and negative ion modes. Raw data were processed in Compound Discoverer 3.3 for peak extraction, alignment, filtering, and normalization. Metabolite identification was carried out using databases including mzCloud, LIPID MAPS, the Human Metabolome Database (HMDB), MoNA, and NIST_2020_MSMS.

### Statistical analysis and visualization

Taxonomic and functional gene abundance data were used to calculate richness and the Shannon diversity index. β-diversity was assessed using Principal Coordinates Analysis (PCoA) based on Bray-Curtis distance, and differences between groups were evaluated using permutational multivariate analysis of variance (PERMANOVA) (64). The Wilcoxon rank-sum test was utilized to assess the significance of differences in diversity indices, taxonomic units, and functional gene feature abundances among groups. The metabolomics differences were visualized using partial least squares discriminant analysis (PLS-DA). All statistical analyses were conducted in R (v4.4.0), and all visualizations were produced using the “ggplot2” package (v3.5.1).

### Conclusion

In this study, we constructed a catalog of 2,474 feline gut microbial genomes and integrated serum metabolomic data to characterize bile acid (BA) transformation and its alteration upon *T. gondii* infection. The results revealed that the feline gut microbiome harbored widespread genetic potential for BA transformation but lacked the complete 7α-dehydroxylation pathway. Furthermore, the specific functions involved in BA transformation differed across intestinal regions. The small intestinal microbiota may play a key role in the oxidation-reduction reactions of the BA 7α-hydroxy group, whereas the large intestinal microbiota appeared to be more inclined to undergo the intermediate steps of BA transformation. Additionally, *T. gondii* infection disrupted BA transformation capacity in the small intestine, supported by increased abundance of BA transformation-related genes and elevated serum cholesterol levels. The findings of this study provided new insights into gut microbiota-associated metabolic perturbations during feline toxoplasmosis.

## Data Availability

Metagenomic sequencing data for this study have been deposited in the National Center for Biotechnology Information (NCBI) under BioProject no. PRJNA1203944. All other data supporting the findings of this study are available in the paper and supplemental material.

## SUPPLEMENTAL MATERIAL

Table S1 aem.00674-26-s0001.xlsx Summary information for 2,474 genomes.

Table S2 aem.00674-26-s0002.xlsx Summary of functional KO profiles and taxonomy of 2,045 bile acid-related genomes.

Table S3 aem.00674-26-s0003.xlsx Summary information of MAGs related to bile acid from humans, pigs, and chickens.

ASM does not own the copyrights to Supplemental Material that may be linked to, or accessed through, an article. The authors have granted ASM a non-exclusive, world-wide license to publish the Supplemental Material files. Please contact the corresponding author directly for reuse.
